# Health literacy competencies among future healthcare providers: a cross-sectional study of preclinical medical students using TSOY-32

**DOI:** 10.1186/s12875-026-03433-z

**Published:** 2026-06-18

**Authors:** Kemal Ozan Lüle

**Affiliations:** Internal Medicine Department, Faculty of Medicine, Gaziantep University, Gaziantep, Türkiye

**Keywords:** Health literacy, Medical students, preclinical education, TSOY-32, Medical education, Health literacy scale

## Abstract

**Background:**

Health literacy is essential for effective physician–patient communication and high-quality healthcare delivery. However, the extent to which future physicians possess these competencies during the early stages of medical training remains insufficiently understood. This study aimed to assess health literacy levels among preclinical medical students using the Turkish Health Literacy Scale-32 (TSOY-32) and to examine differences according to academic year.

**Methods:**

This cross-sectional study was conducted among preclinical medical students (Year 1–3) at Gaziantep University Faculty of Medicine during the 2025–2026 academic year. Health literacy was assessed using the TSOY-32, which provides an overall health literacy index as well as domain- and competence-specific indices. Group comparisons were performed using one-way analysis of variance and chi-square tests. Pearson correlation analyses and multivariable linear regression analyses were performed to evaluate whether academic year was independently associated with overall health literacy after adjustment for sex and body mass index. Sensitivity analyses including age were conducted to assess the robustness of the findings.

**Results:**

A total of 305 students were included. According to the TSOY-32 Overall Health Literacy Index, 74.4% of participants were classified as having inadequate or problematic/limited health literacy. Mean overall health literacy scores differed between academic-year groups (Year 1: 28.51 ± 6.13; Year 2: 28.81 ± 5.82; Year 3: 30.74 ± 6.72; *p* = 0.036), with a small effect size (η² = 0.022). Between-group differences were most apparent for appraisal- and application-related indices. In multivariable analysis adjusted for sex and body mass index, third-year students scored 2.24 points higher than first-year students on the TSOY-32 index (95% CI: 0.47–4.01; *p* = 0.013); however, the model explained only a small proportion of score variability (adjusted R² = 0.022).

**Conclusions:**

A high proportion of students demonstrated suboptimal health literacy (inadequate or problematic/limited). Although third-year students had statistically higher health literacy scores than first-year students, the small effect size and low explained variance indicate that academic year had limited explanatory value. These findings should therefore be interpreted as modest between-group differences rather than evidence of longitudinal progression. As the present study did not directly assess curricular content or educational interventions, these findings are descriptive in nature; whether structured or competency-based educational approaches would improve health literacy in this population remains a hypothesis to be tested in future interventional research.

**Supplementary Information:**

The online version contains supplementary material available at 10.1186/s12875-026-03433-z.

## Introduction

Health literacy is a multidimensional construct that refers to individuals’ abilities to access, understand, appraise, and apply health-related information in order to make informed decisions concerning health and healthcare. In recent years, health literacy has increasingly been conceptualized as a key social determinant of health, influencing health behaviors, use of healthcare services, and health outcomes at both individual and population levels [1, 2]. Limited health literacy has been associated with inadequate engagement in preventive health services, poorer self-management, increased healthcare utilization, and higher rates of adverse health outcomes [2, 3].

Beyond its impact on patients, health literacy has gained growing attention in relation to healthcare professionals and medical education. Effective physician-patient communication, shared decision-making, and patient-centered care require not only clinical expertise but also the ability to convey health information in an understandable and meaningful way. Contemporary evidence highlights that integrating health literacy-oriented competencies into undergraduate medical education may contribute to improved communication skills and safer, more effective healthcare delivery [4, 5].

Medical students represent a particularly important target group for health literacy research, as professional attitudes, communication styles, and health-related perspectives begin to form during the early years of medical training. The preclinical phase of medical education constitutes a critical period in which students acquire foundational biomedical knowledge alongside core competencies related to professionalism, communication, and health promotion. Recent studies suggest that health literacy-related knowledge and skills among medical students may vary across different stages of training, indicating that these competencies do not necessarily develop uniformly throughout medical education [5–7].

In Türkiye, health literacy has been recognized as a public health priority, and validated instruments have been developed to assess health literacy levels in different populations. The Turkish Health Literacy Scale-32 (TSOY-32), developed in alignment with the conceptual framework of the European Health Literacy Survey (HLS-EU), provides a comprehensive assessment of health literacy across the domains of healthcare and disease prevention and health promotion [8]. While the TSOY-32 has been widely applied in various population groups, data focusing specifically on preclinical medical students remain limited, particularly regarding potential differences according to class level.

Therefore, the present study aimed to assess health literacy levels among preclinical medical students using the TSOY-32 and to examine between-year differences in health literacy, including the association between academic year and overall health literacy. By characterizing the distribution of health literacy during the early stages of medical education, this study seeks to provide evidence that may inform curriculum development and support the integration of health literacy-focused educational strategies in undergraduate medical training.

## Materials and methods

### Study design and setting

This cross-sectional study was conducted among preclinical medical students (Year 1–3) enrolled at Gaziantep University Faculty of Medicine during the 2025–2026 academic year.

### Population, sample size, and sampling

The study population consisted of 628 preclinical medical students registered in the faculty during the study period. The primary outcome was the comparison of the TSOY-32 overall health literacy index across class levels. Sample size was calculated using one-way ANOVA assuming a small-to-moderate effect size (Cohen’s f = 0.20), a significance level of α = 0.05, and a statistical power of 80%, resulting in a minimum required sample size of 244 participants; the assumed effect size was informed by recent findings on health literacy levels among medical students reported in the literature [9]. Considering potential missing or incomplete responses, the target sample size was set at approximately 300 students. Participants were recruited using voluntary convenience sampling.

### Inclusion and exclusion criteria

Students were eligible if they:

- were enrolled in the preclinical period (Year 1-3),
- were aged 18 years or older,
- provided informed consent,
- and had sufficient Turkish literacy to complete the questionnaire.

Participants were excluded if they:

- declined participation,
- were younger than 18 years,
- had more than 20% missing responses on the TSOY-32 scale,
- submitted duplicate responses,
- or reported cognitive or language difficulties preventing accurate questionnaire completion.

### Data collection procedure

Data were collected using an anonymous online survey administered via Google Forms. The survey link was distributed through class representatives, and participation was voluntary. No identifying information was collected. Responses were stored in a password-protected electronic database accessible only to the research team. Participants were asked to submit only one response, and duplicate entries were excluded during data cleaning. For clarity, an English translation of the full survey form, including both the general information form and the TSOY-32 items, was provided as a supplementary file. Of the 628 eligible preclinical students enrolled during the study period, 305 provided complete responses, corresponding to a response rate of approximately 48.6%.

### Measures and instruments

Sociodemographic and anthropometric variables: Participants provided information on sociodemographic and anthropometric characteristics, including age (years), sex (male/female), and current academic year (Year 1, 2, or 3). Self-reported height (cm) and body weight (kg) were also collected, from which body mass index (BMI) was calculated using the standard formula of weight in kilograms divided by the square of height in meters (kg/m²). BMI was incorporated into the analyses as an exploratory anthropometric covariate, given prior reports suggesting possible associations between BMI and health-related behaviors, self-management practices, and health information–seeking patterns in young adult populations.

Health literacy assessment: Health literacy was evaluated using the Turkish Health Literacy Scale-32 (TSOY-32). This scale was developed within the framework of the European Health Literacy Survey (HLS-EU) and subsequently adapted into Turkish by Okyay and Abacıgil. The Turkish version was published by the Ministry of Health and has been shown to be a valid and reliable measurement tool, with a reported Cronbach’s alpha coefficient of 0.927, indicating high internal consistency [8].

The TSOY-32 is designed for individuals aged 15 years and older who possess adequate literacy skills. It comprises 32 items that assess health literacy through four fundamental processes: accessing, understanding, appraising, and applying health-related information. These processes are evaluated using a five-point Likert scale with the response options “very easy,” “easy,” “difficult,” “very difficult,” and “I have no idea.”

For analytical purposes, item responses are reverse-coded, and raw scores are converted into standardized index scores ranging from 0 to 50 using the recommended scoring procedure, with higher scores indicating higher levels of health literacy. In addition to the overall health literacy index, TSOY-32 scores were calculated separately as domain-specific indices for healthcare (treatment and services) and disease prevention and health promotion, as well as competence-specific sub-indices reflecting access to information, understanding of information, appraisal of information, and application of information.

Based on established cut-off values, health literacy levels were classified into four categories: inadequate (0–25), problematic/limited (> 25–33), adequate (> 33–42), and excellent (> 42–50). These categories were used for descriptive analyses and categorical comparisons, while continuous index scores were used for parametric and regression analyses [8].

### Statistical analysis

Statistical analyses were performed using IBM SPSS 24.0 Statistics software. Continuous variables were expressed as mean ± standard deviation, and categorical variables as frequencies and percentages. Normality assumptions were assessed using the Shapiro-Wilk test and graphical methods. Comparisons of the TSOY-32 overall health literacy index and predefined health literacy indices across class levels were conducted using one-way analysis of variance (ANOVA). Tukey’s HSD test was applied for post-hoc pairwise comparisons when overall significance was observed. Effect sizes were calculated using eta-squared (η²). Associations between class level and categorical health literacy levels were evaluated using the Chi-square test. Correlations between class level, age, BMI, and health literacy indices were examined using Pearson correlation analysis. A two-tailed p value < 0.05 was considered statistically significant. Multivariable linear regression analysis was performed to evaluate whether academic year was independently associated with overall health literacy after adjustment for sex and body mass index. Academic year was entered into the model using dummy variables with first-year students as the reference category. As a sensitivity analysis, an additional regression model including age was constructed. Multicollinearity was assessed using variance inflation factors (VIFs).

## Results

A total of 305 preclinical medical students were included in the study. The mean age of the participants was 20.72 ± 1.56 years, and the mean body mass index (BMI) was 23.65 ± 4.05 kg/m². Of the participants, 53.1% were male and 46.9% were female. The distribution across academic years was 39.0% first-year, 35.4% second-year, and 25.6% third-year students. According to the TSOY-32 Overall Health Literacy Index classification, 24.6% of the students were categorized as having inadequate health literacy, 49.8% as problematic/limited, 21.3% as adequate, and 4.3% as excellent health literacy (Table 1).

**Table 1:** Characteristics of the study population (*n* = 305)

Variable	*n* (%) / Mean ± SD
Age (years)	20.72 ± 1.56
BMI (kg/m²)	23.65 ± 4.05
Sex	
Male	162 (53.1%)
Female	143 (46.9%)
Class level	
Year 1	119 (39.0%)
Year 2	108 (35.4%)
Year 3	78 (25.6%)
BMI categories	
Underweight	18 (5.9%)
Normal weight	183 (60.0%)
Overweight	84 (27.5%)
Obese	20 (6.6%)
Health literacy level *(TSOY-32 Overall)*	
Inadequate health literacy	75 (24.6%)
Problematic/Limited health literacy	152 (49.8%)
Adequate health literacy	65 (21.3%)
Excellent health literacy	13 (4.3%)

Continuous variables are presented as mean ± standard deviation (SD); categorical variables as n (%)

*BMI* Body mass index, *TSOY-32* Turkish Health Literacy Scale-32

Comparison of TSOY-32 Overall Health Literacy Index scores across academic years showed a statistically significant between-group difference (Year 1: 28.51 ± 6.13; Year 2: 28.81 ± 5.82; Year 3: 30.74 ± 6.72; F = 3.37, *p* = 0.036; η²=0.022) (Table 2). Tukey post-hoc analysis showed that third-year students had higher overall health literacy scores than first-year students (*p* = 0.037). However, the η² value indicated a small effect size, meaning that academic year accounted for only approximately 2.2% of the variance in overall health literacy scores. The corresponding mean difference between Year 3 and Year 1 students (≈ 2.2 points on a 0–50 index scale, representing ~ 4.4% of the scale range) suggests that, although statistically detectable, the practical magnitude of the between-year difference was limited.

**Table 2:** Comparison of TSOY-32 total score and health literacy indices according to class level

Variable	Year 1 Mean ± SD	Year 2 Mean ± SD	Year 3 Mean ± SD	*p*	η²
TSOY-32 Overall Health Literacy Index	28.51 ± 6.13	28.81 ± 5.82	30.74 ± 6.72	**0.036**	0.022
Healthcare (Treatment & Service) domain					
Healthcare - Access Index	31.21 ± 7.86	31.36 ± 7.58	32.14 ± 7.98	0.698	0.002
Healthcare - Understanding Index	30.97 ± 7.71	30.39 ± 7.63	31.87 ± 8.60	0.456	0.005
Healthcare - Appraisal Index	25.11 ± 8.40	24.98 ± 8.86	28.73 ± 8.84	**0.006**	0.034
Healthcare - Application Index	29.33 ± 8.52	30.82 ± 7.70	33.20 ± 7.74	**0.005**	0.035
Disease Prevention / Health Promotion domain					
Health Promotion - Access Index	30.65 ± 8.09	30.51 ± 8.27	31.82 ± 7.96	0.509	0.004
Health Promotion - Understanding Index	30.06 ± 8.79	30.70 ± 8.04	32.08 ± 8.40	0.257	0.009
Health Promotion - Appraisal Index	27.38 ± 7.67	27.71 ± 7.37	29.42 ± 8.88	0.182	0.011
Health Promotion - Application Index	23.37 ± 9.20	24.05 ± 8.56	26.66 ± 8.47	**0.032**	0.022
Process (Competence) Composite Scores					
Access Competence Index	30.93 ± 6.48	30.93 ± 6.69	31.98 ± 6.97	0.494	0.005
Understanding Competence Index	30.51 ± 7.38	30.55 ± 7.10	31.98 ± 7.53	0.324	0.007
Appraisal Competence Index	26.24 ± 7.07	26.34 ± 6.96	29.08 ± 8.22	**0.016**	0.026
Application Competence Index	26.35 ± 7.69	27.44 ± 6.79	29.93 ± 7.33	**0.004**	0.037
Domain Composite Scores					
Healthcare Domain Health Literacy Index	29.15 ± 6.51	29.39 ± 6.13	31.48 ± 6.58	**0.030**	0.023
Health Promotion Domain Health Literacy Index	27.86 ± 6.69	28.24 ± 6.59	29.99 ± 7.52	0.091	0.016

*p* values were obtained using one-way ANOVA. Effect sizes were calculated using eta-squared (η²). *TSOY-32* Turkish Health Literacy Scale-32. Bold values indicate statistical significance (p < 0.05)

When domain-specific and competence-specific indices were examined, significant class-level differences were observed for the Healthcare-Appraisal Index, Healthcare-Application Index, Health Promotion-Application Index, Appraisal Competence Index, Application Competence Index, and the Healthcare Domain Health Literacy Index (all *p* < 0.05). In all significant comparisons, higher scores were predominantly observed among third-year students compared with first-year students (Tables 2 and 3).

Detailed results of indices showing statistically significant differences across academic years are presented in Table 3.

**Table 3:** Health literacy indices showing significant differences according to class level

Index	F	*p*	Significant comparison
Healthcare - Appraisal Index	5.24	**0.006**	Year 3 > Year 1, Year 2
Healthcare - Application Index	5.46	**0.005**	Year 3 > Year 1
Health Promotion - Application Index	3.47	**0.032**	Year 3 > Year 1
Appraisal Competence Index	4.19	**0.016**	Year 3 > Year 1, Year 2
Application Competence Index	5.75	**0.004**	Year 3 > Year 1
Healthcare Domain Health Literacy Index	3.53	**0.030**	Year 3 > Year 1

*p* values were obtained using one-way ANOVA with Tukey post-hoc tests. Bold values indicate statistical significance (p < 0.05)

The distribution of health literacy categories differed significantly across academic years (χ² = 14.11, *p* = 0.028). A higher proportion of students with adequate or excellent health literacy was observed among third-year students (35.9%) compared with first-year (25.2%) and second-year students (18.5%) (Table 4).

**Table 4:** Distribution of health literacy levels according to class level

Health literacy level	Year 1 *n* (%)	Year 2 *n* (%)	Year 3 *n* (%)
Inadequate	37 (31.1%)	23 (21.3%)	15 (19.2%)
Problematic / Limited	52 (43.7%)	65 (60.2%)	35 (44.9%)
Adequate	27 (22.7%)	15 (13.9%)	23 (29.5%)
Excellent	3 (2.5%)	5 (4.6%)	5 (6.4%)

Chi-square: χ² = 14.11, *p* = 0.028

Correlation analysis demonstrated a weak but statistically significant positive correlation between class level and the TSOY-32 Overall Health Literacy Index (*r* = 0.134, *p* = 0.019). Class level also showed weak positive correlations with several indices, including the Healthcare-Appraisal Index, Healthcare-Application Index, Health Promotion-Application Index, Appraisal Competence Index, Application Competence Index, the Healthcare Domain Health Literacy Index, and the Disease Prevention and Health Promotion Domain Health Literacy Index. Age was strongly correlated with class level (*r* = 0.817, *p* < 0.001) but was not significantly associated with the overall health literacy index and showed no consistent significant associations with most health literacy indices. Similarly, BMI was not significantly associated with the TSOY-32 Overall Health Literacy Index and demonstrated no consistent relationship with the majority of health literacy indices (Table 5).

**Table 5:** Correlations of class level, age, and BMI with TSOY-32 indices

Variable	Class level *r* (*p*)	Age *r* (*p*)	BMI *r* (*p*)
TSOY-32 Overall Health Literacy Index	**0.134 (0.019)**	−0.064 (0.265)	−0.074 (0.198)
Healthcare - Access Index	0.045 (0.437)	0.026 (0.657)	−0.107 (0.061)
Healthcare - Understanding Index	0.037 (0.515)	**−0.136 (0.017)**	**−0.165 (0.004)**
Healthcare - Appraisal Index	**0.150 (0.009)**	0.070 (0.226)	−0.021 (0.717)
Healthcare - Application Index	**0.185 (0.001)**	−0.111 (0.052)	0.006 (0.920)
Health Promotion - Access Index	0.092 (0.107)	−0.108 (0.059)	−0.027 (0.633)
Health Promotion - Understanding Index	0.097 (0.090)	0.026 (0.651)	−0.057 (0.318)
Health Promotion - Appraisal Index	**0.141 (0.014)**	0.024 (0.672)	−0.007 (0.903)
Health Promotion - Application Index	0.057 (0.319)	−0.099 (0.084)	−0.112 (0.051)
Access Competence Index	0.073 (0.201)	**−0.136 (0.017)**	−0.105 (0.067)
Understanding Competence Index	**0.141 (0.014)**	0.055 (0.337)	−0.043 (0.455)
Appraisal Competence Index	**0.186 (0.001)**	−0.047 (0.416)	−0.001 (0.986)
Application Competence Index	**0.135 (0.019)**	−0.046 (0.427)	−0.088 (0.124)
Healthcare Domain Health Literacy Index	**0.116 (0.042)**	−0.073 (0.205)	−0.051 (0.375)
Health Promotion Domain Health Literacy Index	**0.134 (0.019)**	−0.064 (0.265)	−0.074 (0.198)

Pearson correlation coefficients are presented as r (p). Statistically significant correlations are shown in bold

In multivariable linear regression analysis adjusting for sex and body mass index, academic year remained significantly associated with overall health literacy. Compared with first-year students, third-year students had 2.24 points higher TSOY-32 scores (B = 2.242, 95% CI: 0.472–4.011, *p* = 0.013), whereas no significant difference was observed between second- and first-year students (B = 0.282, *p* = 0.732). Sex showed a borderline significant association with health literacy, while BMI was not significantly associated. The overall model was statistically significant but explained only a small proportion of the variance in overall health literacy (adjusted R²=0.022, *p* = 0.030), indicating that academic year, sex, and BMI together had limited explanatory value. There was no evidence of multicollinearity (all VIF < 1.3) (Table 6).

**Table 6:** Multivariable linear regression analysis of factors associated with TSOY-32 overall health literacy index

Variable	B	95% CI	*p* value
Sex*	-1.487	-2.961 to -0.012	**0.048**
BMI	-0.562	-1.630 to 0.506	0.301
Year 2 (vs. Year 1)	0.282	-1.333 to 1.896	0.732
Year 3 (vs. Year 1)	2.242	0.472 to 4.011	**0.013**
Adjusted R²=0.022; F(4,300) = 2.722; *p* = 0.030; all VIF < 1.3			

*Reference category: female, academic year was entered as dummy variables with first-year students as the reference category. *BMI* Body mass index. Bold values indicate statistical significance (*p* < 0.05)

In sensitivity analyses including age, academic year remained significantly associated with health literacy, with both second- and third-year students showing higher TSOY-32 scores compared with first-year students. Compared with first-year students, second-year students had 4.36 points higher TSOY-32 scores (B = 4.356, 95% CI: 2.290–6.423, *p* < 0.001), and third-year students had 9.16 points higher scores (B = 9.159, 95% CI: 6.268–12.049, *p* < 0.001). Age was inversely associated with health literacy scores (B=-2.183, *p* < 0.001). The overall model explained 13.2% of the variance in health literacy (R²=0.132, *p* < 0.001), and no problematic multicollinearity was detected (all VIF < 4) (Supplementary Table 1).

## Discussion

Health literacy is increasingly recognized not only as a patient attribute but also as a competency relevant to healthcare professionals responsible for communicating complex medical information within healthcare systems. In this cross-sectional study, health literacy competencies among preclinical medical students—representing future healthcare providers—were assessed using the TSOY-32, and variations according to academic year were examined. The findings indicate that a substantial proportion of students demonstrated inadequate or problematic/limited health literacy, despite being enrolled in a medical education program. Although overall health literacy scores differed across academic years, the small effect size indicates that this difference was limited in practical magnitude. Therefore, the findings should not be interpreted as evidence that academic progression is a strong determinant of health literacy, but rather as a modest between-year difference observed within a cross-sectional sample. Importantly, no established minimum important difference (MID) has been defined for the TSOY-32 in medical student populations. Consequently, a between-group difference of approximately 2 points on a 50-point scale should be considered a statistically detectable but educationally modest signal, which is unlikely to translate into clinically meaningful improvements in patient communication or health information appraisal without additional structured educational input. This interpretive caution is essential to avoid overstating the practical relevance of the observed differences.

Recent studies focusing on medical and health sciences students have similarly reported that limited health literacy is prevalent even in academically advanced populations [10, 11]. These findings support the notion that health literacy extends beyond general education and academic achievement, encompassing context-specific skills related to evaluating and applying health information in real-world health settings [12]. From this perspective, the high proportion of students with suboptimal health literacy may indicate areas within early medical training that could benefit from further structured reinforcement.

The higher overall health literacy scores observed among third-year students may be interpreted in light of previous evidence on health literacy-related competencies in educational contexts [13]. However, given the cross-sectional design and the small effect size, this finding should be interpreted cautiously and should not be taken as evidence of a strong, causal, or educationally substantial effect of academic year.

From a primary care perspective, the observed distribution of health literacy competencies may have direct implications for future physician–patient encounters. Primary care physicians are typically the first point of contact within the healthcare system and routinely manage patients with diverse educational backgrounds, chronic conditions requiring sustained self-management, and complex preventive care needs — all of which depend heavily on the physician’s ability to translate, appraise, and apply health information in patient-centered ways. Limited appraisal and application competencies, which were the most consistently lower domains in our sample, are particularly relevant in primary care settings, where shared decision-making, lifestyle counseling, chronic disease education, and medication adherence support form a substantial part of daily practice. Suboptimal health literacy competencies among future physicians may therefore contribute to miscommunication, low adherence, inadequate preventive care uptake, and inequitable outcomes among patients with limited health literacy. Strengthening these competencies during undergraduate training may not only support individual professional development but may also contribute to safer, more equitable, and more effective primary care delivery. Because our study did not evaluate curricular content or teaching strategies, any inference regarding structured or competency-oriented approaches [14] should be regarded as a potential implication rather than a finding directly supported by the data. The absence of a significant difference between first- and second-year students in the primary model suggests that, in this cross-sectional sample, between-group differences in health literacy were observed primarily when comparing third- and first-year cohorts. Sensitivity analyses including age suggested that the association between academic year and health literacy was not explained solely by age-related variation. Nevertheless, these findings should be interpreted cautiously because age and academic year were strongly correlated, and the cross-sectional design limits conclusions about developmental change.

Domain-specific analyses revealed that class-level differences were most pronounced in appraisal and application-related indices, particularly within the healthcare domain. These competencies are increasingly recognized as essential for clinical reasoning, shared decision-making, and effective patient communication [12, 15]. Recent qualitative and quantitative studies suggest that appraisal and application skills are among the most challenging components of health literacy to develop and may require targeted instructional strategies rather than passive curricular exposure [15, 16]. The pattern observed in the present sample, in which between-group differences were concentrated in these two competence domains, is consistent with this view.

Despite a somewhat higher proportion of adequate or excellent health literacy among third-year students, limited health literacy persisted in a notable subgroup of upper-year students, indicating that higher academic year alone was not consistently associated with adequate competence. Contemporary medical education literature increasingly advocates for the integration of explicit health literacy training during the preclinical years, including communication skills, critical appraisal of health information, and patient education strategies [14]. Similar patterns of limited or heterogeneous health literacy have been reported in medical and healthcare student populations across different educational contexts [17, 18].

Correlation analyses revealed weak but statistically significant associations between class level and several health literacy indices, while age and BMI showed no meaningful relationships with overall health literacy. BMI had been included as an exploratory anthropometric covariate based on prior reports of possible associations with health behaviors and health information–seeking patterns in young adults; however, no such relationship was observed in the present sample, suggesting that anthropometric status may have limited relevance to health literacy in this relatively homogeneous preclinical population. Similar findings have been reported in recent studies involving young adult and student populations, suggesting that demographic and anthropometric factors may play a limited role in shaping health literacy within relatively homogeneous educational settings [11, 14]. After adjusting for age, sex, and BMI, academic year remained a significant independent predictor of health literacy. Interestingly, age showed a negative association when class level was controlled for, suggesting that chronological age alone does not account for the between-group differences in health literacy observed across academic years. It is also possible that older students within the same academic year represent heterogeneous educational trajectories, including delayed entry or alternative academic pathways, which may influence health literacy competencies. However, given the cross-sectional design and the relatively narrow age range of the sample, this observation should be interpreted cautiously.

Overall, the findings suggest that health literacy competencies among preclinical medical students are heterogeneous and that academic year alone explains only a limited proportion of this variability. Rather than indicating a substantial between-group difference attributable to academic year, the results point to the potential value of explicitly evaluating and teaching health literacy-related competencies within undergraduate medical education. Consistent with recent systematic evidence reporting substantial variability in health literacy among university and healthcare student populations [19], our findings highlight the potential value of further investigating whether structured, competency-based health literacy training could strengthen appraisal and application skills in this group. As our cross-sectional design did not assess any educational intervention, the effectiveness of such approaches remains to be determined in future studies.

### Limitations

Several limitations of this study should be acknowledged. First, the cross-sectional design does not allow causal inferences regarding changes in health literacy over time. Although higher health literacy scores were observed among upper-year students, these findings should be interpreted as between-group associations rather than evidence of longitudinal change attributable to medical education.

Second, the study was conducted at a single medical faculty, which may limit the generalizability of the findings to other institutions with different educational structures, curricula, or student profiles. As Turkish medical schools differ in curricular design (traditional, integrated, problem-based, or competency-based) and institutional context, the present results should be considered hypothesis-generating rather than nationally representative. Multicenter studies are needed to confirm reproducibility across diverse educational settings.

Third, health literacy was assessed using a self-reported instrument, which may be subject to response and social desirability biases. Although the TSOY-32 is a validated and reliable tool, self-perceived competencies may not fully reflect actual performance in real-world health literacy tasks, particularly those requiring complex appraisal and application skills. Medical students may be particularly prone to overestimating their competencies due to familiarity with health terminology, and common method bias cannot be excluded as all variables were collected via self-report. Complementing self-report instruments with objective assessments is recommended in future research.

Another important limitation concerns the small effect sizes observed in the study. Although some between-year comparisons and regression coefficients reached statistical significance, the η² value for the overall health literacy comparison and the adjusted R² of the primary regression model were low. Therefore, the findings should be interpreted as statistically detectable but practically modest differences. Academic year should not be considered a strong explanatory factor for health literacy in this sample, and unmeasured individual, educational, social, and motivational factors may account for a substantial proportion of the observed variability. The regression model included only a limited set of covariates (sex and BMI), and unmeasured factors such as parental education, socioeconomic status, prior health-related experience, language proficiency, and individual learning engagement were not available. Residual confounding is therefore likely, and the reported associations should be interpreted as adjusted estimates within a restricted covariate set rather than fully controlled effects.

From a methodological standpoint, age and academic year were strongly collinear in this sample (*r* = 0.817), reflecting the structurally overlapping nature of the two variables in a preclinical cohort. For this reason, age was not included in the primary multivariable model, and the sensitivity model containing both variables should be interpreted with caution despite acceptable VIF values, as high collinearity may inflate standard errors and produce coefficient signs that do not reflect independent effects. Disentangling the contributions of chronological age and cumulative educational exposure would require longitudinal designs in which the two can vary independently.

Finally, participation was based on voluntary convenience sampling, which may have introduced selection bias and limited generalizability. Students with greater interest in health-related topics or greater willingness to participate in surveys may have been more likely to respond, whereas less engaged students may have been underrepresented. With a response rate of approximately 48.6% (305/628) and no data on non-respondents, the true prevalence of inadequate or problematic/limited health literacy in the underlying population may be higher than reported here. Therefore, the observed health literacy distribution may not fully reflect the entire preclinical student population or students in other medical schools with different curricular structures.

## Conclusions

This study found that a substantial proportion of preclinical medical students had inadequate or problematic/limited health literacy despite enrollment in a medical education program. Overall health literacy scores differed statistically across academic years, with higher scores among third-year students than first-year students; however, the effect size was small and the primary regression model explained only a limited proportion of score variability. Therefore, academic year should be interpreted as a modest correlate rather than a strong explanatory factor for health literacy. These findings suggest academic year alone is not strongly associated with health literacy in this preclinical sample. As the cross-sectional design precludes inference about change over time, no conclusion can be drawn regarding the adequacy of current curricular exposure; this question requires longitudinal evaluation. As the present study did not evaluate curricular content or educational interventions, any inference regarding optimal instructional strategies remains hypothesis-generating. Longitudinal and interventional studies are therefore needed to determine whether structured, competency-based health literacy training can produce educationally meaningful improvements, particularly in appraisal and application skills.

## Supplementary Information

Supplementary Material 1.

Supplementary Material 2.

## Data Availability

The datasets generated and/or analyzed during the current study are not publicly available due to institutional data protection policies but are available from the corresponding author on reasonable request.

## Abbreviations

ANOVA: Analysis of Variance
BMI: Body Mass Index
CBME: Competency-Based Medical Education
CI: Confidence Interval
HL: Health Literacy
HLS-EU: European Health Literacy Survey
SD: Standard Deviation
TSOY-32: Turkish Health Literacy Scale-32
VIF: Variance Inflation Factor
