# Supplementary material for: Health literacy competencies among future healthcare providers: a cross-sectional study of preclinical medical students using TSOY-32
Source: BMC Prim Care. 2026 Jun 18;27:275. doi: 10.1186/s12875-026-03433-z (PMC13401316; doi:10.1186/s12875-026-03433-z)
Supplement: Supplementary file 1 — Supplementary Material 1. [file 12875_2026_3433_MOESM1_ESM.docx]

**Supplementary Table 1. Sensitivity analysis including age**

| **Variable** | **B** | **95% CI** | **p** |
| --- | --- | --- | --- |
| Age | -2.183 | -2.924 to -1.441 | **<0.001** |
| Sex* | -1.465 | -2.865 to -0.064 | **0.040** |
| BMI | -0.521 | -1.536 to 0.494 | 0.313 |
| Year 2 (vs Year 1) | 4.356 | 2.290 to 6.423 | **<0.001** |
| Year 3 (vs Year 1) | 9.159 | 6.268 to 12.049 | **<0.001** |
| Adjusted R²=0.118; F(5,299)=9.117; **p<0.001** , all VIF<4 | | | |

*Sensitivity analysis including age. Reference category: female. Academic year was entered as dummy variables with first-year students as the reference category. BMI = body mass index. Bold values indicate statistical significance (p < 0.05).*
