# Supplementary material for: Health literacy competencies among future healthcare providers: a cross-sectional study of preclinical medical students using TSOY-32
Source: BMC Prim Care. 2026 Jun 18;27:275. doi: 10.1186/s12875-026-03433-z (PMC13401316; doi:10.1186/s12875-026-03433-z)
Supplement: Supplementary file 2 — Supplementary Material 2. [file 12875_2026_3433_MOESM2_ESM.docx]

**English Translation of the Survey Form Used in the Study**

Note: This document presents an English translation of the survey form used in the study for reviewer and reader convenience. The original Turkish version was used during data collection. The English version is provided for informational purposes and should not be considered a separately validated instrument.

**Part 1. General Information Form**

Please answer the following questions.

1. **Age:** ______ years
2. **Sex:**
   - Female
   - Male
3. **Academic year / period:**
   - Period 1
   - Period 2
   - Period 3
4. **Height:** ______ cm
5. **Weight:** ______ kg

**Part 2. Turkish Health Literacy Scale-32 (TSOY-32) – English Translation**

Below are statements about various health-related issues.
For each statement, please indicate the degree of difficulty by marking the option that best applies to you:
**very easy / easy / difficult / very difficult / don’t know**

| **No.** | **If you were to rate from very easy to very difficult, how easy or difficult would it be for you to do the following?** | **Very easy** | **Easy** | **Difficult** | **Very difficult** | **Don’t know** |
| --- | --- | --- | --- | --- | --- | --- |
| 1 | When you have a complaint about your health, find out whether it may be a sign of illness |  |  |  |  |  |
| 2 | When you have a complaint about your health, read and understand written materials about it (such as brochures, booklets, or posters) |  |  |  |  |  |
| 3 | When you have a complaint about your health, evaluate whether advice from your family or friends on this issue is reliable |  |  |  |  |  |
| 4 | When you want to visit a healthcare institution, find out which doctor you should consult |  |  |  |  |  |
| 5 | When you want to visit a healthcare institution, find out how to make your application (such as scheduling an appointment) |  |  |  |  |  |
| 6 | When you want to visit a healthcare institution, make an appointment by telephone or via the internet |  |  |  |  |  |
| 7 | Find information about the treatments of diseases that concern you |  |  |  |  |  |
| 8 | Understand your doctor’s explanations about your illness |  |  |  |  |  |
| 9 | Evaluate the advantages and disadvantages of different treatment options recommended by your doctor |  |  |  |  |  |
| 10 | Use your medications in the way recommended by healthcare professionals (such as doctors or pharmacists) |  |  |  |  |  |
| 11 | Understand the instructions for using the medication written on the medicine package |  |  |  |  |  |
| 12 | Decide whether you need to seek a second opinion from another doctor |  |  |  |  |  |
| 13 | Understand information about preparations required before laboratory tests/examinations (such as following a diet) |  |  |  |  |  |
| 14 | Find the location of the unit you want to reach in the hospital (such as the laboratory or outpatient clinic) |  |  |  |  |  |
| 15 | Decide what you can do in an emergency situation (such as an accident or sudden health problem) |  |  |  |  |  |
| 16 | Call an ambulance when necessary |  |  |  |  |  |
| 17 | Have your regular health follow-ups and check-ups performed at the intervals recommended by your doctor |  |  |  |  |  |
| 18 | Find information about conditions that may be harmful to your health, such as being overweight or having high blood pressure |  |  |  |  |  |
| 19 | Understand health warnings related to conditions that may be harmful to your health, such as being overweight or having high blood pressure |  |  |  |  |  |
| 20 | Find information about how to cope with unhealthy behaviors such as smoking or insufficient physical activity |  |  |  |  |  |
| 21 | Understand health warnings about how to cope with unhealthy behaviors such as smoking or insufficient physical activity |  |  |  |  |  |
| 22 | Find information about health screenings you should undergo according to your age, sex, and health status (such as breast screening for women or prostate-related screening for men) |  |  |  |  |  |
| 23 | Understand recommendations in sources such as the internet, newspapers, television, or radio about what should be done to be healthier |  |  |  |  |  |
| 24 | Decide whether recommendations in sources such as the internet, newspapers, television, or radio about what should be done to be healthier are reliable |  |  |  |  |  |
| 25 | Understand information on food packaging that you think may affect your health |  |  |  |  |  |
| 26 | Evaluate the positive and negative characteristics of your environment (such as your home, street, or neighborhood) that affect health |  |  |  |  |  |
| 27 | Find information about what can be done to make your environment (such as your home, street, or neighborhood) healthier |  |  |  |  |  |
| 28 | Evaluate which of your daily behaviors (such as exercising, eating healthy, or not smoking) affect your health |  |  |  |  |  |
| 29 | Change your lifestyle (such as exercising, eating healthy, or not smoking) for the sake of your health |  |  |  |  |  |
| 30 | Be able to follow a written diet plan provided by a dietitian |  |  |  |  |  |
| 31 | Make suggestions to your family or friends about being healthier |  |  |  |  |  |
| 32 | Interpret changes in health-related policies |  |  |  |  |  |
